# The psychometric properties of the Swahili version of the Primary Care Post Traumatic Stress Disorder screen for DSM-5 among adults in Kenya

**DOI:** 10.3389/fpsyt.2024.1338311

**Published:** 2024-09-03

**Authors:** Patrick N. Mwangala, Joseph Newton Guni, Paul Mwangi, Millicent Makandi, Anita Kerubo, Rachel Odhiambo, Amina Abubakar

**Affiliations:** ^1^ Institute for Human Development, Aga Khan University, Nairobi, Kenya; ^2^ Centre for Geographic Medicine Research Coast, Kenya Medical Research Institute (KEMRI), Kilifi, Kenya; ^3^ School of Public Health, University of the Witwatersrand, Johannesburg, South Africa; ^4^ Department of Psychiatry, University of Oxford, Warneford Hospital, Oxford, United Kingdom

**Keywords:** factor analysis, Swahili-PC-PTSD-5, adults, Kenya, psychometric properties

## Abstract

**Background:**

The psychometric properties of the Primary Care PTSD Screen for DSM-5 (PC-PTSD-5) are undocumented in Kenya and sub-Saharan Africa (SSA) at large. This study aimed to evaluate the psychometric properties of the Swahili version of the tool, S-PC-PTSD-5, in a community sample of adults 18 years and older drawn from Nairobi, Mombasa and Kwale counties in Kenya.

**Methods:**

Analysis of cross-sectional data from 1431 adults from the community was conducted, examining the reliability, factorial structure, measurement invariance, and convergent and divergent validity of the interviewer-administered S-PC-PTSD-5.

**Results:**

Out of 1431 adults who completed the S-PC-PTSD-5, 666 (46.5%) reported experiencing at least one traumatic event. Internal consistency of the S-PC-PTSD-5 was good overall, with alpha and omega values above 0.7. Confirmatory factor analysis (CFA) results indicated a one-factor structure of the S-PC-PTSD-5 for the overall sample. Multigroup CFA also demonstrated factorial invariance for sex for the one-factor structure of S-PC-PTSD-5. Scores for S-PC-PTSD-5 significantly correlated (positively) with those of generalized anxiety disorder (GAD7) and depressive symptoms (PHQ9), indicating convergent validity. S-PC-PTSD-5 scores also significantly correlated (negatively) with the WHO-5 wellbeing index, supporting divergent validity.

**Conclusions:**

The S-PC-PTSD-5 is a reliable and valid unidimensional measure. It appears to be a valuable screening measure for probable PTSD in both urban and rural community settings in Kenya. Nonetheless, to confidently identify those who may need treatment/additional support, further research on the reliability and validity of S-PC-PTSD-5 is required, especially its diagnostic accuracy at different cutoff scores.

## 1 Introduction

Post-traumatic stress disorder (PTSD) is a common psychiatric condition that arises from experiencing or witnessing traumatic events that involve actual or threatened death, serious injury, or sexual violence (1–3). PTSD involves four clusters of symptoms relating to: re-experiencing symptoms of a distressing event (e.g., intrusive thoughts), hyperarousal (e.g., irritability), avoidance of any reminders of the distressing event, and negative moods or cognitions associated with the event (4). According to the World Health Organization (WHO), about 70% of people around the world will experience a potentially traumatic event during their lifetime (5). However, only a minority (about 6%) will go on to develop PTSD (6). Biological mechanisms play an important role in determining risk and resilience (7). The incidence of PTSD varies across populations, countries and trauma types (8–11), with estimates suggesting about 4% of the global population experience PTSD at some point in their lifetime (6). Individuals living in sub-Saharan Africa (SSA) are disproportionately exposed to trauma and may be at a heightened risk for PTSD. Repeated and extended exposure to armed conflict, mass-casualty events, and violence, combined with a large treatment gap, may result in a significant effect on the population burden of PTSD in SSA (12). About 80% of people with PTSD in low- and middle-income countries (LMICs) do not receive treatment (6). Little research has examined the predictors of the long-term course of PTSD. The extant literature suggests that even though a significant proportion of cases recover within a few months, at least a third of the cases persist for many years (13–15) and that chronic PTSD can lead to secondary disorders (16) and suicidality (17). People who experience PTSD often experience comorbid psychiatric conditions, including depression and anxiety. About 30-50% of persons with PTSD also have major depression (18). Similarly, the co-occurrence of PTSD with anxiety is high (19). The comorbidity of PTSD with depression and anxiety is problematic because such people demonstrate greater symptom severity (20), show a more chronic course of impairment (21), and have poorer treatment outcomes and an increased risk of dropout (22). Individuals experiencing PTSD also report reduced quality of life, and poorer functional outcomes (23, 24). PTSD also incurs a high economic burden, both direct and indirect costs (25, 26). On average, about 3.6 working days per month are lost on account of PTSD (6, 27). Therefore, early diagnosis and intervention are critical for effective treatment and reducing the long-term outcomes associated with PTSD.

In the past few decades, several assessment tools for trauma and related symptoms have been developed due to increased interest in identifying and screening PTSD (28). The Primary Care Post-traumatic Stress Disorder Screen (PC-PTSD-5) is the most up-to-date five-item measure that is short and easy to administer (29, 30). It comprises five items requiring “yes” or “no” responses. It is identical to the PC-PTSD, except for the revised trauma screening question and the addition of a fifth item (31). The trauma exposure screening question was designed such that individuals who do not report trauma exposure do not answer subsequent questions about PTSD symptoms, thus preventing unnecessary administration of the remaining items. The fifth item was added to assess the new symptom cluster of negative alterations in mood and cognitions, particularly guilt and blame.

Several studies have used the PC-PTSD-5 measure in SSA among different populations, including adolescents receiving ART in South Africa (32), young people in Uganda, South Africa and Zimbabwe (33), maternal healthcare providers in Malawi (34), healthcare workers during the early phase of COVID-19 pandemic in Kenya (35), communities affected by the COVID-19 pandemic in Uganda (36), hospital sample of adults in Mozambique (37) and persons with HIV in Uganda (38). Nonetheless, to the best of our knowledge, there is no record of the reliability and validity of the PC-PTSD-5 tool among the existing studies despite its growing popularity in the region. The lack of data on PC-PTSD-5 adaptation and validation in SSA is an important impediment to PTSD research in the region, e.g. in the accurate assessment of PTSD burden, evaluating the efficacy of PTSD psychological interventions, and determining program cost-effectiveness. Outside SSA, PC-PTSD has been adapted and validated in a host of populations including veterans (39, 40), family members of healthcare workers during the COVID-19 pandemic (41), medical staff exposed to the COVID-19 pandemic (42), college students (43), substance misusing and trauma-exposed adults (29, 30), PTSD patients, non-PTSD patients and healthy controls (31), children with traumatic exposure (44), adolescents in pediatric primary care, civilian primary care adults (45), and firefighters (46). However, there is hardly any validation report involving community-based individuals. Taken together, most of the extant PC-PTSD-5 adaptation and validation literature emanates from the United States of America, and most of these studies have examined the predictive validity of the screen. Notably, the majority of the existing studies have not considered other aspects of validity and reliability. The current study fills this gap by reporting the reliability (internal consistency) and validity (divergent, convergent and construct) of the interviewer-administered S-PC-PTSD-5 screen among adults 18 years and older from urban and rural informal settlements across Kenya.

## 2 Materials and methods

### 2.1 Study setting, design, and participants

Data used in this study was obtained from the formative phase of the ‘Advancing Gender Equality through Civil Society’ (AGECS) mental health research project in Kenya. The AGECS project is an ongoing mixed methods research study being implemented in the urban informal settlements of Nairobi and Mombasa counties and the rural setting of Kwale county in Kenya. Kwale and Mombasa are located on the Kenyan coast, and Nairobi is Kenya’s capital city. The main objective of the AGECS project is to evaluate the burden of mental health problems among women and their spouses and then design, implement, and assess the impact of mental health interventions in this population. The study is being conducted sequentially in three phases, namely formative, design and implementation phases. The formative phase of the AGECS project was conducted in 2023. It comprised several activities, including systematic reviews, a cross-sectional survey, qualitative explorations and an assessment of the mental health systems. The data being reported in this paper was obtained from the cross-sectional survey component of the AGECS formative phase.

The target population in the cross-sectional survey was adult men and women aged 18 years and older living in the three counties of interest, i.e. Nairobi. Mombasa and Kwale. Potential clients were recruited using sequential sampling from households in the three counties. Participant recruitment went on until the desired sample size was attained. Recruitment of participants was conducted by community health volunteers (CHVs) at the household level. We drew on the CHVs’ knowledge of both the geography and local population of the study sites to identify potential clients. To be included in the survey, participants had to be at least 18 years old, be able to provide informed consent, and be able to speak Swahili or English. Most of the assessments were carried out in Swahili. Once recruited by CHVs, participants were booked for assessment, usually the following day, at a central venue in the community, which included social halls, churches and mosques. All clients who turned up on the day of assessment were consented to the study by a team of 11 trained research assistants who then conducted different sets of assessments that included information on sociodemographic, economic background, and mental health. All data was collected face-to-face using the Open Data Kit (ODK) through tablets that were password-protected and encrypted to avoid data loss. A data manager double-checked any inconsistencies in the data before uploading it to the server daily. After the assessment, a small refreshment was provided to the participants. Costs incurred, such as travel to attend assessments, were also reimbursed to the participants.

**Figure 1:**
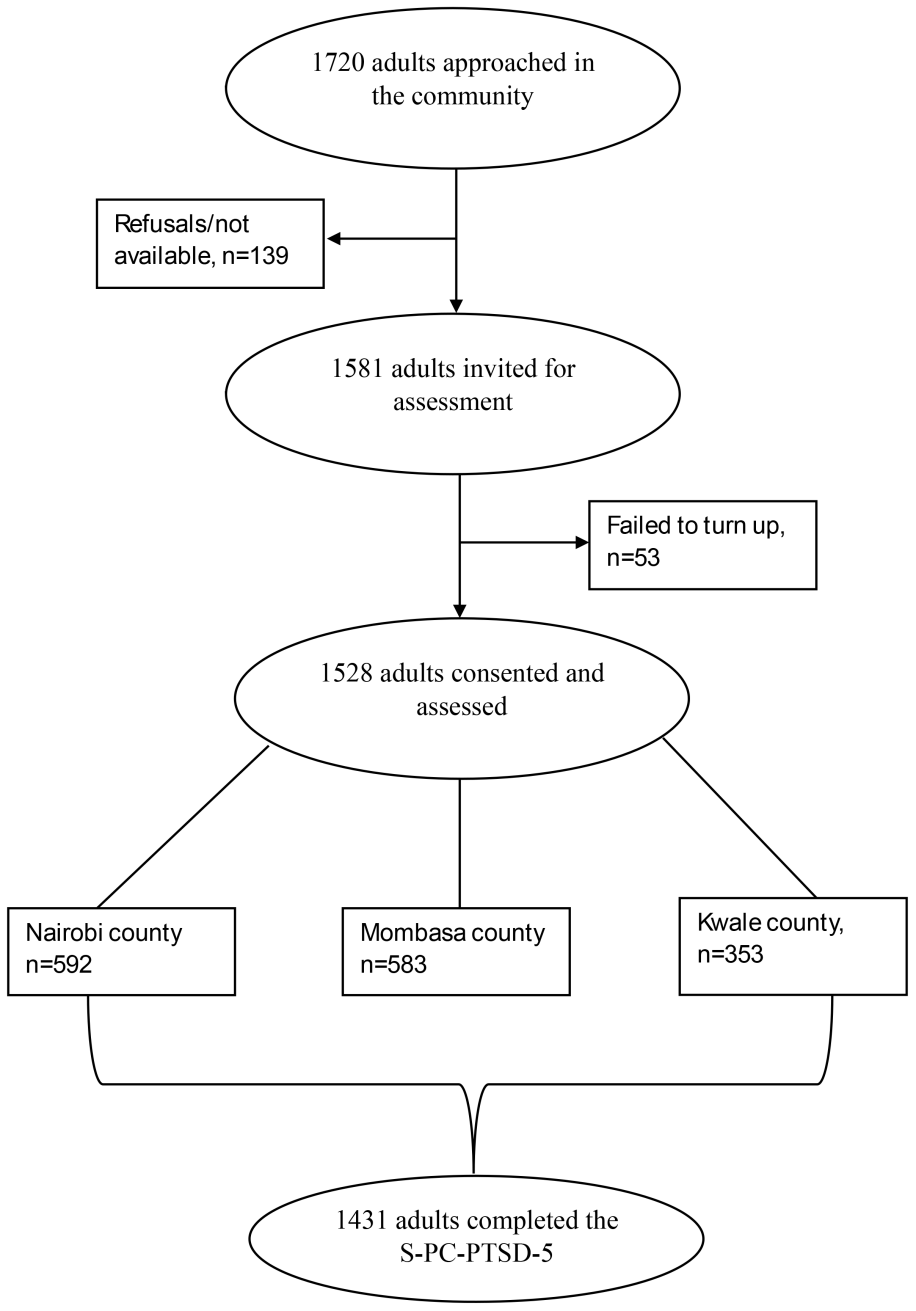
Flowchart of participant recruitment.

One thousand seven hundred and twenty adults were approached by CHVs to participate in the study. Of those, 139 refused to take part in the project for different reasons, e.g. unavailability. An additional 53 did turn up on the day of the assessment. A total of 1528 participants were then assessed across the three counties (353 from Kwale, 583 from Mombasa, and 592 from Nairobi). Among participants who completed the survey, 1431 completed the S-PC-PTSD-5 measure and were included in the statistical analysis. Details are highlighted in **Figure 1**.

### 2.2 Measures relevant to the present analysis

#### 2.2.1 Sociodemographic characteristics

A sociodemographic questionnaire capturing data on participant age, sex, level of education, employment, religion, and marital status was administered by the research assistants in a face-to-face interview.

#### 2.2.2 Depressive symptoms

We used the 9-Item Patient Health Questionnaire (PHQ-9) to assess for depressive symptoms (47). It consists of nine items that assess how often the client has been bothered by the following problems over the last two weeks: i) ‘little interest or pleasure in doing things’; ii) ‘feeling down, depressed or hopeless’; iii) ‘trouble falling or staying asleep, or sleeping too much’; iv) ‘feeling tired or having little energy’; v)’ poor appetite or overeating’; vi) ‘feeling bad about yourself or that you are a failure or have let yourself or your family down’; vii) ‘trouble concentrating on things, such as reading the newspaper or watching television’; viii) ‘moving or speaking so slowly that other people could have noticed or the opposite being so fidgety or restless that you have been moving around a lot more than usual; and ix) ‘thoughts that you would be better off dead or hurting yourself.’ These items are scored on a 4-point Likert scale (0= not at all, 1= several days, 2 = more than a week, 3 = nearly every day). PHQ-9 has been validated in several African countries (48–50). In the Kenyan context, the validation of the PHQ-9 in the Swahili language has been done extensively, and there is empirical evidence that it is suitable for use among diverse populations, including adolescents (51–53), adults living with HIV (54) and healthcare providers (55).

#### 2.2.3 Anxiety

We used the 7-Item Generalized Anxiety Disorder Questionnaire (GAD7) to assess for anxiety symptoms. It consists of seven items that assess how often the client has been bothered by the following problems over the last two weeks: i) ‘feeling nervous, anxious or on edge’; ii) ‘not being able to stop or control worrying’; iii) ‘worrying too much about different things’; iv) ‘trouble relaxing’; v) ‘being so restless that it is hard to sit still’; vi) ‘becoming easily annoyed or irritated’ and vii) ‘feeling afraid as if something awful might happen.’ These items are scored on a 4-point Likert scale (0= not at all, 1= several days, 2 = more than a week, 3 = nearly every day) (56). GAD7 has previously been validated in East Africa and shown to have good psychometric properties (55, 57, 58).

#### 2.2.4 Post-Traumatic Stress Disorder (PTSD)

PC-PTSD-5 is designed to screen for PTSD in primary care settings (39). Respondents first complete a single question to determine lifetime trauma exposure. Individuals who deny exposure to a specific factor are assigned a score of zero. In contrast, those who acknowledge exposure are asked five questions: i) ‘In the past month, have you had nightmares about the event(s) or thought about the event(s) when you did not want to?’; ii) ‘In the past month, have you tried hard not to think about the event(s) or went out of your way to avoid situations that reminded you of the event(s)?’; iii) ‘In the past month, have you been constantly on guard, watchful, or easily startled?’; iv) ‘In the past month, have you felt numb or detached from people, activities, or your surroundings?’ and v) ‘In the past one month, have you felt guilty or unable to stop blaming yourself or others for the event(s) or any problems the event(s) may have caused?’ The five items are scored dichotomously (0=no; 1=yes) regarding the presence of post-traumatic stress disorder (PTSD) symptoms experienced during the preceding month. The total scores can vary from 0 to 5 (40). Before being used in the study, the PC-PTSD-5 underwent translation into Swahili in line with international guidelines for translation of tools in health research (59). The measure was independently translated from English to Swahili by two staff members who were fluent in both languages. After that, back-translation into English was done by another independent pair of translators. A panel of Kenyan researchers, knowledgeable about the culture and fluent in both English and Swahili and the translators held a harmonization meeting to ensure conceptual, content, semantic and idiomatic equivalence of the tool.

#### 2.2.5 Wellbeing

The WHO-5 wellbeing index is a self-administered psychological wellbeing measure (60, 61). It consists of five items: i) ‘I have felt cheerful and in good spirits’; ii) ‘I have felt calm and relaxed’; iii) ‘I have felt active and vigorous’; iv) ‘I woke up feeling fresh and rested’; and v) ‘My daily life has been filled with things that interest me.’ These items positively assess the degree of wellbeing during the past two weeks. They are scored on a 6-point Likert scale whereby ‘0’ stands for (at no time) and ‘5’ for (at all times). WHO-5 Swahili version has been previously validated in Kenya, and it has been observed to retain its good psychometric properties (61).

### 2.3 Ethical clearance

The primary project was approved by the Aga Khan, Nairobi Institutional Scientific and Ethics Review Committee (Ref: 2022/ISERC_44(V2)). Permission to conduct the study in Kenya was granted by the National Commission for Science, Technology, and Innovation (Ref: 346643). Local permit to conduct the study was granted by the research office in Nairobi (Ref: NCCG/DHS/REC/240), Mombasa (Ref: MCG/COPH/RCH./111) and Kwale (Ref: CG/KWL/6/5/1/CECM/39/VOL.1/34). All participants provided written informed consent for their participation.

### 2.4 Statistical analysis

We analyzed our data using R statistical software version 4.1.2 (62). Sociodemographic information was summarized using descriptive statistics: frequency and proportion for categorical variables and mean and standard deviation for continuous variables. Internal consistency reliability for S-PC-PTSD-5 was computed using Cronbach’s alpha (α) and Macdonald’s omega (ω) (63). Alpha and omega values ≥0.7 are considered to show good internal consistency.

Construct validity, the extent to which a measure, S-PC-PTSD-5, assess the underlying construct it is supposed to measure, was examined using confirmatory factor analysis (CFA) (64). CFA is a statistical technique that seeks to confirm if the number of factors (or constructs) and the loadings of observed (indicator) variables on them conform to what is expected on the basis of theory (64). Before conducting CFA, we checked for multivariate normality, outliers and missing data. There were no outliers and missing data, and the S-PC-PTSD-5 items were normally distributed. Additionally, we used the Kaiser-Meyer-Olkin (KMO) test for sampling adequacy to evaluate dataset appropriateness before conducting CFA. A value of KMO estimate above 0.7 was deemed acceptable (65). Bartlett’s test of sphericity was also performed to assess the adequacy of the data for CFA. In order to examine CFA model’s goodness of fit, a number of statistics were used: root mean square error of approximation (RMSEA), standardized root mean square residual (SRMSR), comparative fit index (CFI), and Tucker-lewis index (TLI) (66). An RMSEA value of <0.08 was deemed an acceptable fit, and <0.05 was considered a good fit, while an SRMR value of <0.06, CFI and TLI values of >0.95 demonstrated an excellent fit (66). We used unweighted least squares (ULS) model estimators because they are distribution-free and yield consistent estimates (67). Measurement of invariance was utilized to assess whether the S-PC-PTSD-5 had an invariant one factor across sex (females vs. males). To do this, a sequence of invariance models: configural, metric, and scalar invariance models, were tested. Subsequently, the invariance models were compared, metric versus configural and scalar versus metric using CFI and a CFI change of ≤0.01 demonstrated the unidimensionality of the S-PC-PTSD-5. The lavaan package was used to compute CFA in R (68).

Convergent validity, the degree to which different methods measuring the same trait yield similar results, was measured by correlating the S-PC-PTSD-5 scores with generalized anxiety (GAD7) and depressive symptoms (PHQ9). On the other hand, divergent validity was assessed by correlating S-PC-PTSD-5 scores with WHO-5 wellbeing index scores. Divergent validity examines whether constructs that should have no relationship do, in fact, not have any relationship. Spearman correlation coefficients were used to evaluate convergent and divergent validity of S-PC-PTSD-5 because the S-PC-PTSD-5 total scores were not normally distributed. Values of <0.3, 0.3 to 0.5, and above 0.5 indicated weak, moderate, and robust correlation, respectively. For all hypothesis tests, a two-tailed p-value <0.05 was deemed statistically significant.

## 3 Results

### 3.1 Sample characteristics

**Table 1** presents the sociodemographic characteristics of the 1431 study participants. Of the total, 905 (63.2%) participants were from the coast of Kenya (Kwale and Mombasa counties), and 526 (36.8%) were from Nairobi. The average age of the participants was 39.5 years (SD=13.2), ranging from 18 to 94. Over half of the participants were female (n=984, 68.8%), Christian (n=891, 62.3%), and married (n=1061, 74.1%). Among the married individuals, the majority reported having a monogamous marriage (n=1005, 94.7%). Almost three-quarters of the participants reported having a nuclear family structure (n=1065, 74.4%). Most participants were married and having children, 1331 (93%). 205 (14.3%) reported earning a living from skilled employment.

**Table 1:** Participants’ sociodemographic characteristics.

		Overall (N=1431)
**Age in years**	Mean (SD)	39.5 (13.2)
**Study location**	Coast	905 (63.2%)
	Nairobi	526 (36.8%)
**Sex**	Male	447 (31.2%)
	Female	984 (68.8%)
**Religion**	Christian	891 (62.3%)
	Islam	537 (37.5%)
	Traditional	2 (0.1%)
	Other	1 (0.1%)
**Marital status**	Never married	90 (6.3%)
	Cohabiting	10 (0.7%)
	Married	1061 (74.1%)
	Separated/divorced	181 (12.7%)
	Widowed	84 (5.9%)
	Other	5 (0.3%)
**Marriage type (*for those married*)**	Monogamous	1005 (94.7%)
	Polygamous	56 (5.3%)
**Family type**	Nuclear	1065 (74.4%)
	Extended	234 (16.4%)
	Single parent	124 (8.7%)
	Other	8 (0.5%)

### 3.2 The item distribution of the S-PC-PTSD-5

**Table 2** summarizes the item distribution for the S-PC-PTSD-5. A total of 1431 participants used the S-PC-PTSD-5. Among them, 666 (46.5%) reported experiencing at least one of the following events: a serious accident, physical or sexual assault, earthquake or flood, witnessing injury or death of others, and the homicide or suicide of a loved one. Among the 666 participants, nearly half reported experiencing nightmares (n=326, 48.8%). Over half reported trying hard not to think about the events (n=428, 64.3%) and feeling constantly on guard or easily startled (n=396, 59.5%). Additionally, 165 participants (24.8%) reported feeling numb or detached from people, activities, and their surroundings. More than a quarter of the participants felt guilty or could not stop blaming themselves or others for the events (n=190, 28.5%).

**Table 2:** Distribution of S-PC-PTSD-5 item responses across the sample (n=1431).

PC-PTSD-5 items (only for those who experienced the above events)	Yes n(%)	No n(%)	Alpha if the item is dropped	Corrected total item correlation
**1. Had nightmares about the event(s) or thought about the event(s) when you did not want to?**	326 (48.9%)	340 (51.1%)	0.71	0.61
**2. Tried hard not to think about the event(s) or went out of your way to avoid situations that reminded you of the event(s)?**	428 (64.3%)	238 (35.7%)	0.70	0.63
**3. Been constantly on guard, watchful, or easily startled?**	396 (59.5%)	270 (40.5%)	0.70	0.62
**4. Felt numb or detached from people, activities, or your surroundings?**	165 (24.8%)	501 (75.2%)	0.73	0.54
**5. Felt guilty or unable to stop blaming yourself or others for the event(s) or any problems the event(s) may have caused?**	190 (28.5%)	476 (71.5%)	0.71	0.62
The Internal consistency				
** Cronbach’s Alpha [95% CI]**	0.75 [0.72 - 0.78]			
** Macdonald’s Omega [95% CI]**	0.76 [0.73 - 0.78]			

### 3.3 Internal consistency of the S-PC-PTSD-5 scale

**Table 2** displays the internal consistency results for the S-PC-PTSD-5. The findings indicate that the scale demonstrated good internal consistency, with a Cronbach’s Alpha of 0.75 and Macdonald’s Omega of 0.76.

### 3.4 Construct validity

**Figure 2:**
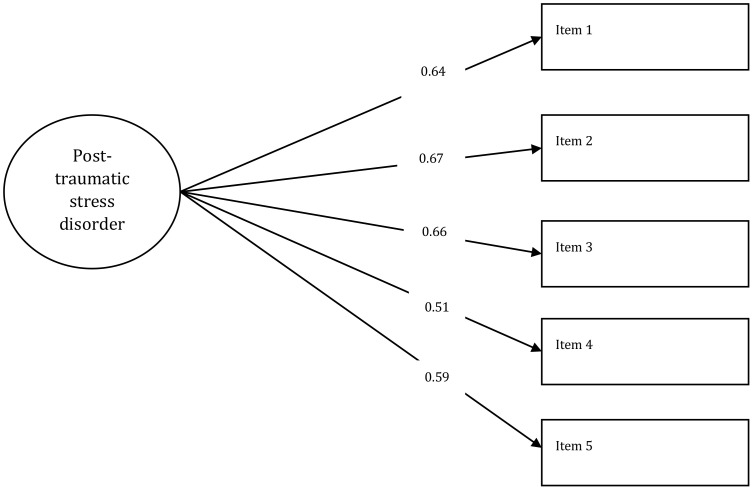
The unidimensional CFA model with the factor loadings. The circle represents the post-traumatic stress disorder construct, and the rectangles represent the PC-PTSD-5 scale items. The error variances for Item 1, Item 2, Item 3, Item 4, and Item 5 were 0.15, 0.13, 0.14, 0.14, and 0.13, respectively.

KMO and Bartlett’s test of sphericity results, 0.75 and χ2 (10, n=666) = 757.44, p < 0.001), respectively, confirmed that our data was adequate for CFA. **Table 3** presents the results of the CFA and measurement invariance analysis across sex for the S-PC-PTSD-5 scale. The CFA model exhibited excellent fit indices, including RMSEA = 0.000, SRMR = 0.066, TLI = 1.00, and CFI = 1.00, supporting the unidimensionality of the S-PC-PTSD-5 scale. All factor loadings were significant and ranged between 0.51 and 0.67, surpassing the threshold of 0.35 (64, 69), indicating a strong relationship between the post-traumatic stress disorder construct and the items (**Figure 2**).

**Table 3:** The CFA and Measurement invariance across males versus females (n=666).

	RMSEA (90% CI)	SRMR	TLI	CFI	ΔCFI
**Unidimensional factor**	0.000 (0.000 – 0.027)	0.066	1.000	1.000	-
Measurement invariance (males vs females)					
**Configural invariance**	0.000 (0.000 – 0.000)	0.059	1.000	1.000	-
**Metric invariance**	0.000 (0.000 – 0.000)	0.063	1.000	1.000	<0.01
**Scalar invariance**	0.000 (0.000 – 0.000)	0.066	1.000	1.000	<0.01

Additionally, the measurement invariance was conducted to assess whether the unidimensionality of the S-PC-PTSD-5 scale was invariant across sex. First, in a model assuming the same item-factor assignment (configural invariance), the one-factor solution of S-PC-PTSD-5 fitted the data well across participants’ sex (males and females), as shown in **Table 3**. Second, the Metric invariance, after constraining the factor loadings to be the same across sex, indicated excellent fit indices (**Table 3**). Lastly, the Scalar invariance after constraining the intercepts and factor loadings for the same S-PC-PTSD-5 scale item to be equal across sex had an excellent fit index (**Table 3**). The change in CFI for configural invariance versus metric invariance and metric invariance versus scalar invariance was less than 0.01, which is below the recommended threshold of 0.01. This finding indicates that the unidimensionality of the S-PC-PTSD-5 scale is generalizable across males and females.

### 3.5 Convergent and divergent validity

**Table 4** summarizes the divergent and convergent validity of the PC-PTSD-5. The results indicate that the S-PC-PTSD-5 showed a significant negative correlation with the WHO-5 wellbeing index (r=-0.21, p <0.001), supporting the expected negative association between post-traumatic stress disorder and wellbeing. This finding demonstrates the divergent validity of the scale.

**Table 4:** The Convergent and Divergent validity (n=666).

	Convergent	Divergent
**Wellbeing (WHO-5)**		-0.21 (p <0.001)
**Depression (PHQ-9)**	0.40 (p <0.001)	
**Generalized anxiety (GAD-7)**	0.38 (p <0.001)	

In contrast, the post-traumatic stress disorder construct was significantly positively correlated with depression (r=0.40, p <0.001) and generalized anxiety (r=0.38, p <0.001), indicating a positive association. This result supports the convergent validity of the PC-PTSD-5, as it demonstrates that the scale correlates positively with related constructs such as depression and anxiety.

## 4 Discussion

### 4.1 Summary of project findings

Valid and reliable screening tools are essential for early diagnosis and mitigation of PTSD. Unfortunately, studies validating measures of PTSD in SSA are limited. The Primary Care PTSD Screen for DSM-5 (PC-PTSD-5) is one of the most commonly utilized PTSD screens in research and practice globally, given its brevity and ease of administration. Although initially designed to identify people with probable PTSD in primary care settings, it has also been used in multiple at-risk populations. In the current study, we sought to investigate the internal consistency reliability, construct, divergent and convergent validity of the interviewer-administered S-PC-PTSD-5 screen among adults 18 years and older from the general population. Overall, we found that the S-PC-PTSD-5 had good internal consistency. The PC-PTSD-5 also retained a unidimensional latent structure where all items loaded well on a one-factor solution, and this model was invariant across sex. The screen also presented good convergent and discriminant validity. To the best of our knowledge, this is the first study in SSA to examine the reliability and validity of the PC-PTSD-5.

### 4.2 Internal consistency

We employed two statistical approaches to assess the internal consistency of the S-PC-PTSD-5, the conventional Cronbach’s alpha and Macdonald’s omega. The latter is believed to give more accurate reliability estimates (70, 71). The reliability coefficients were identical with both methods, demonstrating that the S-PC-PTSD-5 is internally consistent. Our findings are consistent with what has been reported elsewhere among substance misusing, trauma-exposed, socioeconomically vulnerable adults in the US (30), trauma-exposed patients in the US (29), PTSD patients, non-PTSD patients and healthy controls in South Korea (31), medical staff exposed to COVID-19 pandemic (42) and family members of Chinese healthcare workers during the COVID-19 pandemic (41). Test-retest data of the S-PC-PTSD-5 was not collected in this study. Future studies can explore this to consolidate the reliability of the tool further.

### 4.3 Convergent and divergent validity

The significant and positive correlation between S-PC-PTSD-5 with depression and generalized anxiety scores provided evidence of convergent validity. This finding corroborates findings from previous validation studies, albeit in different populations (41, 42, 44). Huang and colleagues found a strong positive correlation between the Chinese version of the tool (C-PC-PTSD-5) and the Post-traumatic Stress Disorder Checklist for DSM-5 (PCL-5), which indicated good convergent validity among medical staff exposed to COVID-19 (42). Still in China, Cheng and colleagues found that the PC-PTSD-5 had significant correlations with the PCL-5 but weak correlations with measures of generalized anxiety, depression and perceived stress, suggesting strong evidence of convergent validity (41). We also found a significant negative correlation between PC-PTSD-5 scores and the WHO-5 wellbeing index, supporting the expected negative association between post-traumatic stress and wellbeing, thus demonstrating the divergent validity of the screen. Our findings on divergent validity are similar to those reported in a previous study in China (41).

### 4.4 Construct validity

Among the few validation studies of the PC-PTSD-5 globally (30, 31, 39–46, 72), this is the first study to report on the factorial structure and measurement invariance of the PC-PTSD-5. A vast majority of the existing studies have focused on documenting the performance characteristics of the PC-PTSD-5, that is, sensitivity (ability of the test to detect a true positive), specificity (ability of the test to detect a true negative), and receiver operating characteristics (overall diagnostic performance of the test in comparison with other tests), in addition to its reliability and various validity aspects (31, 39, 40, 44, 46). The majority of the existing studies on PC-PTSD-5 recommend a cutoff score of ≥3 as optimal for a positive probable PTSD screen (31, 39, 40, 44, 46). In the current study, we observed that the S-PC-PTSD-5 is a unidimensional scale evidenced by good item factor loadings and excellent goodness of fit indices to a pre-specified one-factor structure. Regarding measurement invariance, we observed that the unidimensional S-PC-PTSD-5 can be generalized across participant sex (females and males). We are unaware of past studies that have explored these constructs to compare or contrast our findings. More studies are required to substantiate our findings.

### 4.5 Implications

Screening for PTSD is a critical step in the provision of good preventative standard of care. This is especially important for people residing in SSA because they are disproportionately exposed to trauma and the huge treatment gap for PTSD in this setting. Even though there are a number of PTSD screening tools available, the PC-PTSD-5 has demonstrated good psychometric properties in previous research. Our preliminary findings in this study extend the PC-PTSD-5 for potential use among adults in the community. However, further replication and extension (e.g. establishing the diagnostic accuracy of the tool) should be considered before the clinical rollout of the measure. Generally, universal screening programs are rarely recommended, except when the screening strategy is very cheap, the screening measure has high accuracy, the consequences for being a false positive are minimal, and the consequences of failing to diagnose are grave (that is, there is a highly effective and cost-effective intervention and a very poor outcome without the treatment). Despite its popularity in epidemiological research in Kenya, the PC-PTSD-5 would require further study before it can be rolled out in primary care as an adequate screener for PTSD. There is a need to establish its diagnostic properties against gold standard measures, as well as examine its clinical utility, e.g. which cadre of healthcare providers will administer the tool and how they will be trained, and establish a care pathway for those identified to be having PTSD. We also recommend further epidemiological studies to identify known risk factors of PTSD in Kenya and use these to develop a risk score and only screen those with a higher risk score (this will increase the diagnostic accuracy of the tool).

### 4.6 Strengths and limitations

To our knowledge, this is the first study in SSA and among the few available studies worldwide to comprehensively investigate the psychometric properties of the PC-PTSD-5 screen. Our study extends the evidence on the reliability and validity of the PC-PTSD-5 screen by focusing on an under-investigated area – factorial structure and measurement invariance using a relatively large sample size from three Kenyan settings. However, we did not examine the diagnostic accuracy of the S-PC-PTSD-5; thus, we recommend additional studies in similar settings to explore the sensitivity and specificity of the tool to identify the optimal cutoff score for identifying those who may need treatment or referral services.

### 4.7 Conclusion

The current study investigated the reliability and validity of the S-PC-PTSD-5 among adults aged 18 years and older from the general population in three Kenyan settings. Our findings demonstrate that the PC-PTSD-5 has good psychometric properties and can be used as a reliable, valid, and time-saving tool to assess PTSD. Its general acceptability and utilization in the community and broader population spectrums will identify those with PTSD, facilitating early intervention and delivery of mental health care.

## Data Availability

The raw data supporting the conclusions of this article will be made available by the authors, without undue reservation.
